# Lactate trafficking inhibition restores sensitivity to proteasome inhibitors and orchestrates immuno‐microenvironment in multiple myeloma

**DOI:** 10.1111/cpr.13388

**Published:** 2023-02-15

**Authors:** Alessandro Barbato, Cesarina Giallongo, Sebastiano Giallongo, Alessandra Romano, Grazia Scandura, Saoca Concetta, Tatiana Zuppelli, Marco Lolicato, Giacomo Lazzarino, Nunziatina Parrinello, Vittorio Del Fabro, Paolo Fontana, M'hammed Aguennoz, Giovanni Li Volti, Giuseppe A. Palumbo, Francesco Di Raimondo, Daniele Tibullo

**Affiliations:** ^1^ Department of Clinical and Experimental Medicine University of Catania Catania Italy; ^2^ Department of Medical, Surgical Sciences and Advanced Technologies G.F. Ingrassia University of Catania Catania Italy; ^3^ Department of General Surgery and Medical‐Surgical Specialties University of Catania Catania Italy; ^4^ Division of Hematology AOU Policlinico Catania Italy; ^5^ Department of Clinical and Experimental Medicine University of Messina Messina Italy; ^6^ Department of Molecular Medicine University of Pavia Pavia Italy; ^7^ Departmental Faculty of Medicine and Surgery UniCamillus‐Saint Camillus International University of Health and Medical Sciences Rome Italy; ^8^ IOM Ricerca Srl Viagrande Italy; ^9^ Department of Biomedical and Biotechnological Sciences, Section of Biochemistry University of Catania Catania Italy

## Abstract

Metabolic changes of malignant plasma cells (PCs) and adaptation to tumour microenvironment represent one of the hallmarks of multiple myeloma (MM). We previously showed that MM mesenchymal stromal cells are more glycolytic and produce more lactate than healthy counterpart. Hence, we aimed to explore the impact of high lactate concentration on metabolism of tumour PCs and its impact on the efficacy of proteasome inhibitors (PIs). Lactate concentration was performed by colorimetric assay on MM patient's sera. The metabolism of MM cell treated with lactate was assessed by seahorse and real time Polymerase Chain Reaction (PCR). Cytometry was used to evaluate mitochondrial reactive oxygen species (mROS), apoptosis and mitochondrial depolarization. Lactate concentration resulted increased in MM patient's sera. Therefore, PCs were treated with lactate and we observed an increase of oxidative phosphorylation‐related genes, mROS and oxygen consumption rate. Lactate supplementation exhibited a significant reduction in cell proliferation and less responsive to PIs. These data were confirmed by pharmacological inhibition of monocarboxylate transporter 1 (MCT1) by AZD3965 which was able to overcame metabolic protective effect of lactate against PIs. Consistently, high levels of circulating lactate caused expansion of Treg and monocytic myeloid derived suppressor cells and such effect was significantly reduced by AZD3965. Overall, these findings showed that targeting lactate trafficking in TME inhibits metabolic rewiring of tumour PCs, lactate‐dependent immune evasion and thus improving therapy efficacy.

## Graphical abstract

One of the hallmarks of multiple myeloma (MM) is metabolic changes of malignant plasma cells (PCs) and adaptation to tumour microenvironment (TME). The metabolic reprogramming of MM cells shapes TME towards a hypoxic, acid and nutrient depleted niche, thereby supporting tumour proliferation and immune evasion. Malignant PCs are known to secrete lactate and we previously demonstrated that mesenchymal stromal cells from MM patients are more glycolytic than same cells from healthy donors. Hence, we aimed to explore the impact of high lactate concentration on cellular metabolism of tumour PCs and efficacy of the proteasome inhibitors.

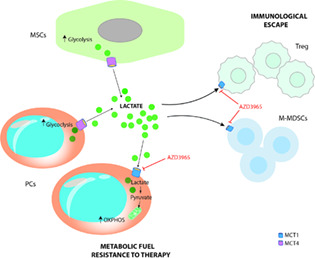

## 1 INTRODUCTION

Multiple myeloma (MM) is a haematological malignancy characterized by the presence of abnormal clonal plasma cells (PCs) in bone marrow (BM) causing hypercalcemia, renal failure, anaemia and bone lesions, kidney injury and hypercalcemia. As a result of the advantages in therapeutic strategies, there has been a great improvement in the outcome of MM in the last decade.^1^ However, it remains an incurable disease as patients eventually develop relapsed or refractory MM.^2^ The progression from the early stages of the disease as monoclonal gammopathy of undetermined significance (MGUS) and smouldering MM (SMM) to MM is drastically affected by the tumour microenvironment (TME).^3^ Indeed, it is well established that TME acts as a determinant player in tumour cell growth, adaptation and resistance to anti‐cancer therapy.^4^, ^5^, ^6^, ^7^ In this context, malignant PCs rapidly reprogram their metabolism to adapt to cellular stress, prevent apoptosis and escape immune system.^8^ Concomitantly, cancer metabolic reprogramming reshapes TME towards a hypoxic, acid (due to high lactate concentration) and nutrient depleted niche, thereby supporting tumour proliferation and immune evasion.^9^ Moreover, compared to healthy PCs, which shift from glycolysis to oxidative phosphorylation (OXPHOS) during differentiation into antibody‐secreting cells,^10^ malignant PCs rely on both increased glycolytic rate and OXPHOS.^7^ It has been demonstrated that the glycolysis rate‐limiting enzyme hexokinase 2 (HK2) is highly expressed in newly diagnosed MM (NDMM) patients, it is increased along disease progression, and it is associated with poor prognosis.^11^, ^12^ Furthermore, also lactate dehydrogenase A (LDHA) is highly expressed in MM cells^13^ and it is one of the prognostic factors predicting for MM adverse outcomes.^14^ In addition to glycolysis, MM cells also rely on mitochondrial metabolism as indicated by the induction of cell death after inhibition of both OXPHOS and the glucose uptake.^15^ Consistently, glutamine dependency has been demonstrated upon inhibition of glycolysis and OXPHOS is mainly fuelled by glutaminolysis.^16^ This metabolic plasticity of MM cells is their strength but also represents a vulnerability to be targeted. Among drugs extensively used for MM treatment, proteasome inhibitors (PIs) target cancer cell metabolism by affecting the homeostasis between protein synthesis, folding and destruction.^17^ Additionally, PIs trigger changes in mitochondrial integrity.^18^, ^19^ Therefore, when OXPHOS is forced, myeloma PCs become resistant to PIs.^17^, ^20^, ^21^, ^22^

The metabolic plasticity of MM cells is potentiated by TME which confers metabolic privileges to cancer cells by increasing the availability of glucose and glutamine and by excessive lactate production. Glycolytic cancer cells and cancer associated fibroblasts are the main producers of lactate in the tumour niche.^23^ We recently demonstrated that mesenchymal stromal cells (MSCs) from MM patients are more glycolytic and produce more lactate than healthy counterpart.^24^ Lactate is transported across the plasma membrane by proton‐linked monocarboxylate transporters (MCTs) such as MCT1 (mainly lactate import) and MCT4 (mainly lactate export).^25^ MM cells are able to take up exogenous lactate through MCT1 and it has been demonstrated that inhibition of this incorporation causes apoptosis.^26^ For a long time, lactate was only recognized as a metabolic waste product; however, it has now been demonstrated that it can be used as fuel for oxidative phosphorylation^26^, ^27^ and that it can even act as an oncometabolite with signalling properties. Lactate is sensed by the G protein‐coupled receptor GPR132 of macrophages leading to cancer cell adhesion, migration and invasion correlating with metastasis and poor prognosis in breast cancer.^28^ Tumour cells highly express lactate receptor GPR81,^29^ which acts as an extracellular lactate sensor regulating genes involved in lactate uptake and metabolism.^30^ The loss of GPR81 reduces MCT levels and decreases the growth of cancer cells.^29^, ^31^ In addition to metabolic fuel, the largely produced lactate in TME is used as an important immune suppressor by cancer cells. Indeed, lactate inhibits lymphocytes activity eventually driving the polarization of macrophages towards a M2‐like phenotype.^32^ So overall, it is known that high concentrations of lactate indicate a bad prognosis for survival in cancer patients.^32^

Here, we demonstrated that high lactate concentration in TME increases mitochondrial metabolism of malignant PCs culminating into the reduction of sensitivity to PIs, concurring to support of immune escape mechanisms. We demonstrated that lactate‐mediated resistance and immune evasion are dependent on its metabolic reprogramming and not on the activation of a lactate/GPR81‐mediated signalling.

## 2 MATERIAL AND METHODS

### 2.1 Cell cultures and treatments

Human myeloma cell lines (HMCLs) were cultured in RPMI 1640 medium supplemented with 10% (OPM2, NCI–H929) or 20% (U266) FBS and 1% penicillin/streptomycin at 37°C and 5% CO_2_. After written informed consent (Azienda Ospedaliero‐Universitaria Policlinico “G.Rodolico‐San Marco”, n. 54/2022/PO), peripheral blood mononucleated cells (PBMCs) were obtained from healthy donor buffy coat after separation by Ficoll‐Hypaque gradient. Cocultures of MM cell lines with PBMCS were set using a 2:1 PCs/PBMCs ratio. PBMC medium was supplemented with 5 mg/ml phytohemagglutinin (PHA) for Treg evaluation.

Bortezomib (15 nM) and carfilzomib (3 nM) were obtained from Sigma‐Aldrich (St. Louis, MO). Used doses of Sodium lactate, 3‐hydroxybutyric acid (3‐OBA), 3,5‐Dihydroxybenzoic acid (3,5 DHBA) (Sigma‐Aldrich) and AZD3965 (Astra Zeneca, Mylan, Italy) were respectively, 20 mM, 3 mM, 150 μM and 10 μM.

### 2.2 Lactate concentration measurement

Spectrophotometric determination of lactate was carried out using an Agilent 89090A spectrophotometer (Agilent Technologies, Santa Clara, CA) as previously described.^33^ Briefly, the reaction mixture contained 100 mM Tris–HCl, 1.5 mM N‐ethyl‐N‐2‐hydroxy‐3‐sulfopropyl‐3‐methylalanine, 1.7 mM 4‐aminoantipyrine and 5 IU horseradish peroxidase. Fifty microliters of serum or deproteinized MM cells (3 × 10^6^) for intracellular determination of lactate were added to the mixture, let to stand for 5 min and the absorbance at 545 nm wavelength was recorded. The reaction was started with the addition of 5 IU of lactate oxidase to the cuvette (finale volume = 1 ml) and it was considered ended when no change in absorbance was recorded for at least 3 min. To calculate lactate in serum samples, the difference in absorbance at 545 nm wavelength (Δabs) of each sample was interpolated with a calibration curve obtained by plotting Δabs measured in standard solutions of lactate with increasing known concentrations.

### 2.3 qPCR

After RNA extraction, reverse transcription was performed by using the high‐capacity cDNA Reverse Transcription Kit (Thermo Fisher Scientific, Milan, Italy). Then the relative transcription of human genes MCT1 (Fw: TGTTGTTGCAAATGGAGTGT Rw: AAGTCGATAATTGATGCCCATGCCAA), MCT4 (Fw: TATCCAGATCTACCTCACCAC Rw: GGCCTGGCAAAGATGTCGATGA), GPR81 (Fw: TTCGTATTTGGTGGCAGGCA Rw:TTTCGAGGGGTCCAGGTACA), HK2 (Fw: ATGAGGGGCGGATGTGTATCA Rw: GGTTCAGTGAGCCCATGTCAA), topoisomerase 1 (TPI‐1; Fw: ATGGCTGAAGTCCAACGTCT Rw: AAGGAAGCCATCCACATCAG), enolase 1 (ENO‐1; Fw: AAAGCTGGTGCCGTTGAGAAG Rw: AGCATGAGAACCGCCATTGAT), enolase 2 (ENO‐2; Fw: TGCCTCAGAGTTTTATCGTG Rw:CTTGAGCAGCAGACAGTTG), pyruvate kinase 1 (PKM1; Fw: CGAGCCTCAAGTCACTCCAC Rw: GTGAGCAGACCTGCCAGACT), pyruvate kinase 2 (PKM2; Fw: ATTATTTGAGGAACTCCGCCGCCT Rw: ATTCCGGGTCACAGCAATGATGG), LDHA (Fw: GGATCTCCAACATGGCAGCCTT Rw: AGACGGCTTTCTCCCTCTTGCT), ATP syntase (ATPsynt; Fw: AGCTCAGCTCTTACTGCGG Rw: GGTGGTAGTCCCTCATCAAACT) Cytocrome B (CytB; Fw: TCCTCCCGTGAGGCCAAATATCAT Rw:AAAGAATCGTGTGAGGGTGGGACT) and mitochondrially encoded NADH:ubiquinone oxidoreductase core subunit 4 (MT‐ND4; Fw: ACAAGCTCCATCTGCCTACGACAA; Rw: TTATGAGAATGACTGCGCCGGTGA) was determined by RTqPCR using Brilliant III Ultra‐Fast SYBR Green QPCR Master Mix (Agilent Technologies, Milan, Italy) and 7900HT Fast Real‐Time PCR System (Thermo Fisher Scientific). For each sample, the relative expression level of the mRNA of interest was determined by comparison with the control housekeeping gene B2M (Fw: AGCAGCATCATGGAGGTTTG; Rw: AGCCCTCCTAGAGCTACCTG) using the 2^−ΔΔCt^ method.

### 2.4 Western blot

For Western blot analysis 50 μg of protein was loaded onto a 12% polyacrylamide gel Mini‐ PROTEAN TGXTM (BIO‐RAD, Milan, Italy) followed by electrotransfer to nitrocellulose membrane Trans‐Blot TurboTM (BIO‐RAD, Mylan, Italy) using Trans‐Blot SE Semi‐Dry Transfer Cell (BIO‐RAD). Subsequently, membrane was blocked in chemiluminescent blocker (Millipore, Darmstadt, Germany) for 1 h at room temperature. After blocking, the membrane was three times washed in phosphate‐buffered saline (PBS) for 5 min and incubated with primary antibodies against human MCT1 (ab90582, Abcam, Mylan, Italy), MCT4 (ab234728, Abcam), and β‐actin (ab181602, Abcam). Next day, after three washes in TBST, the membranes were incubated with antimouse (1:3000, Jackson, WestGrove, PA) and anti‐rabbit HRP‐conjugated (1:3000, Jackson, WestGrove, PA) secondary antibodies for 1 h at RT. Proteins bands were visualized with premixed ready‐to‐use chemiluminescent HRP detection reagent (Millipore) according to the manufacturer's instructions and captured using the C‐DiGit Blot Scanner (LI‐COR Biosciences, NE). The density of each band was quantified using ImageJ analysis software and normalized β‐actin levels measured in the same membranes.

### 2.5 Flow cytometry

Mitochondrial reactive oxygen species (mROS) were detected using the MitoSOX™ Red mitochondrial superoxide indicator (ThermoFisher Scientific, Milano, Italy). Cells were stained using 3 μM of the MitoSOX™ Red mitochondrial superoxide indicator (ThermoFisher Scientific, Milano, Italy). After incubation for 10 min at 37°C, cells were washed three times and analysed by using flow cytometry (MACSQuant Analyzer 10, Miltenyi Biotec).

To evaluate apoptosis after drug treatment, cells were stained with annexin AV FITC/7‐ADD assay kit (Beckman Coulter, Mylan, Italy) according to the manufacturer's instructions. Evaluation of apoptosis was performed by flow cytometry. Samples were washed and resuspended in 100 μl of PBS. A 1 μl of Annexin V‐FITC solution and 5 μl of propidium iodide were added to cell suspension and mixed gently. Cells were incubated for 15 min in the dark. Finally, 400 μl of 1× binding buffer was added and cell preparation was analysed by flow cytometry.

A membrane potential probe, the 3,3′‐diethyloxacarbocyanine iodide (DiOC2(3), Thermo Fisher Scientific), was used to evaluate the mitochondrial membrane potential. Cells were incubated with 10 μM DiOC2(3) for 30 min at 37°C, washed twice, resuspended in PBS and analysed by flow cytometry through the detection of the green fluorescence intensity of FITC.

For Treg analysis, immune cells were stained with CD4‐PEVio770 (clone SFCI12T4D11), CD25‐APC (clone B1.49.9) and FOXP3‐PE (clone 259D), all from Beckman Coulter, and Treg were defined as CD4^+^CD25^high^Foxp3^+^. For monocytic myeloid derived suppressor cells (M‐MDSCs) analysis, immune cells were stained with CD14‐FITC (clone 322A.1, TLY4) and HLA‐DR‐APC (clone Immu‐357), all from Beckman Coulter, and M‐MDSCs were defined as CD14^+^HLA‐DR^−^.

### 2.6 Seahorse analysis

Live cell analysis of oxygen consumption rate (OCR) was measured using Seahorse Extracellular Flux Analyzer XF24 (Seahorse Bioscience/Agilent, Milan, Italy). Cells were cultured in the XF24‐well plate overnight at 100.000 cells per well after wells polylysination and allowed to seed overnight. For analysis, assay media was prepared similar to culture media (25 mM glucose, 1 mM sodium pyruvate, and 4 mM l‐glutamine) and pH was adjusted to 7.4 ± 0.1. Manufacturer's protocol was followed for the Cell Mito Stress Test kit with port B containing oligomycin (ATP‐Synthase inhibitor) at 1.0 μM, port C with 1.5 μM FCCP (mitochondrial membrane depolarizer), and port D with a mixture of 0.5 μM of each rotenone (complex I inhibitor) and anti‐mycin A (complex III inhibitor) (final well concentrations). For experiments evaluating the acute response to lactate in MM cells, Port A on the XFp cartridge was used for injection of culture medium (control) or 20 mM Lactate. Seahorse XF‐24 Wave software was used to analyse the data and OCR detection was represented as pmoles/min. All Seahorse data were normalized to the total number of cells and counted by nuclear DAPI staining following the assay.

### 2.7 Cell proliferation

Cell growth was determined using EVE™ automated cell counter (NanoEntek, Seoul Korea, Republic of South Korea). Viable and dead cells were distinguished by tripan blue exclusion. At least three independent counts were done on each sample and three independent experiments were performed.

### 2.8 Immunofluorescence assay

Paraffin sections from biopsy specimens from resistant/refractory MM patients (*n* = 3) and early‐stage MM patients (*n* = 4) were de‐paraffinized and rehydrated as previously described.^34^ Sections were permeabilized using 0.3% Triton X and blocked to prevent non‐specific antibody binding using a 0.3% Triton X‐10% NGS solution. The slides were then incubated overnight at 4°C with the primary antibodies mouse anti‐MCT1 (Abcam) and rabbit anti‐CD138 (Abcam) at 1:100 dilution in 0.3% Triton‐X. Subsequently, cells were washed three times in PBS for 5 min and then incubated for 1 h at room temperature with the appropriate combination of fluorescence conjugated secondary antibodies donkey polyclonal anti‐rabbit Alexa Fluor 647 (A32849, Thermo Fisher Scientific; 1:500) and subsequently with goat polyclonal anti‐mouse Alexa Fluor 488 (A21247, Thermo Fisher Scientific 1:500). Samples were then washed in 0.3% Triton X in PBS and nuclei were counterstained with DAPI (1:1000) for 5 min, at room temperature. Slices were mounted with fluorescent mounting medium Permafluor (Thermo Fisher Scientific) and digital images were acquired using a Zeiss Axio Imager Z1 Microscope with Apotome 2 system (Zeiss, Milan, Italy).

### 2.9 Statistical analysis

All statistics were performed using GraphPad Prism (version 5.00 for Mac, GraphPad Software, San Diego, CA). All data were tested for normality using Shapiro–Wilk test. Data that passed normality test were statistical analysed using Student's *t*‐test or ANOVA test where appropriate. A *p*‐value <0.05 was considered to indicate a statistically significant difference between experimental and control groups.

## 3 RESULTS

### 3.1 Patients with plasma cell disorders show high levels of circulating lactate

**FIGURE 1:**
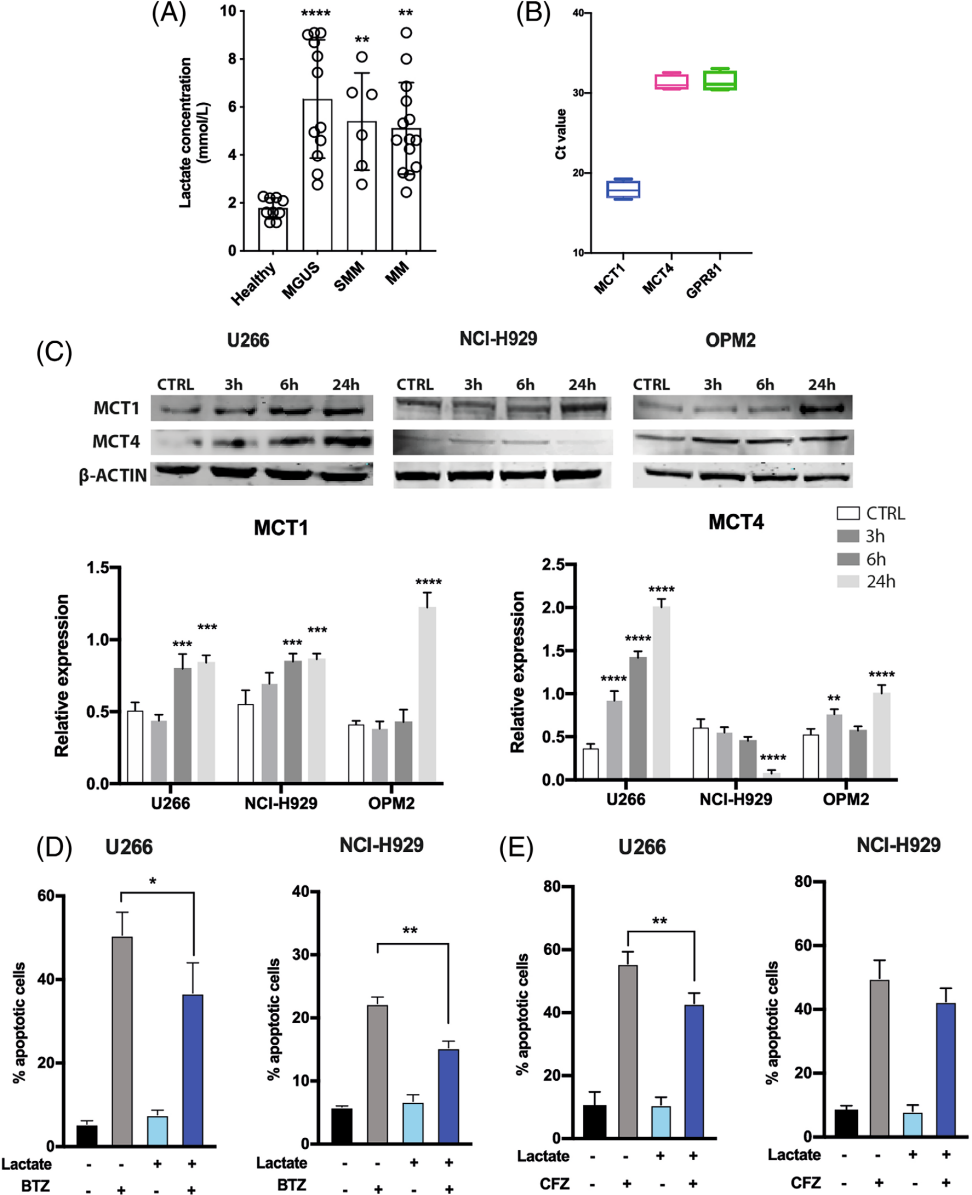
Lactate concentration in patients' sera and lactate transporters in human myeloma cell lines (HMCLs) analysis. (A) Evaluation of the amount of circulating lactate in peripheral blood from patients with plasma cell disorders in respect with healthy controls. (B) Boxplot for the Ct values of MCT1, MCT4 and GPR81 from RT‐qPCR analysis of HMCLs (U266, NCI‐H929 and OPM2). (C) Western blot analysis of MCT1 and MCT4 proteins after 3, 6 and 24 h lactate exposure. β‐Actin protein was used as total protein loading reference. For analysis of Western blot, the optical density of the bands was measured using Scion Image software. (D, E) Quantification of apoptotic cells upon BTZ and CFZ exposure in multiple myeloma cell lines grown in medium control or supplemented with lactate for 72 h. All the data are presented as means ± *SD* of three independent experiments. *p < 0.05; **p < 0.01; ***p < 0.001; ****p < 0.0001

Lactate concentration was measured in peripheral blood (PB) sera of MGUS (*n* = 12), SMM (*n* = 6) and MM at diagnosis (*n* = 14) compared to healthy matched controls (*n* = 9). The amount of lactate was significantly higher in sera from patients with PC disorders compared to healthy controls (6.3 ± 2.5 in MGUS, 5.4 ± 2 in SMM and 5.1 ± 1.9 in MM patients vs. 1.8 ± 0.4 in controls; *p* < 0.0001, *p* < 0.01 and *p* < 0.01 respectively; Figure 1A). No difference was found comparing patients at the different stages of disease. The increased levels of lactate found in patients' sera did not correlate with serum LDH‐A concentration (*p* = 0.98) (Figure S1A).

To investigate the effects of high lactate concentration on MM cells, we first evaluated the expression of lactate transporters, MCT1 and MCT4, and its receptor GPR81 in HMCLs. MCT1 showed higher levels of expression in all tested MM cell lines (cycle threshold [Ct] value: 17.9 ± 1) compared to the other genes which exhibited lower levels of expression (Ct values: 31.2 ± 0.9 for MCT4 and 31.4 ± 1.2 for GPR81; Figure 1B). We therefore evaluated the expression of MCT1 and MCT4 following exposure to high lactate concentration. HMCLs increased expression of MCTs, although at different times (Figure 1C), except for NCI‐H929 cells which showed a significant decrease of MCT4 after 24 h. Also changes in GPR81 mRNA expression were observed after 3 h lactate exposure in all tested HMCLs (Figure S1B).

Next, the effect of lactate on the PI‐induced apoptosis was investigated. U266 and NCI‐H929 cells cultured in medium with lactate for 72 h showed a reduction of the percentage of BTZ‐induced apoptosis (of about 13.8 ± 1.7% and 7 ± 1% respectively; *p* < 0.05 and *p* < 0.01; Figure 1D). The sensitivity to CFZ was reduced by lactate only in U266 cells (12.6 ± 0.4% less than control; *p* < 0.01; Figure 1E).

### 3.2 Blocking lactate import increases PI‐induced cytotoxicity in myeloma PCs

**FIGURE 2:**
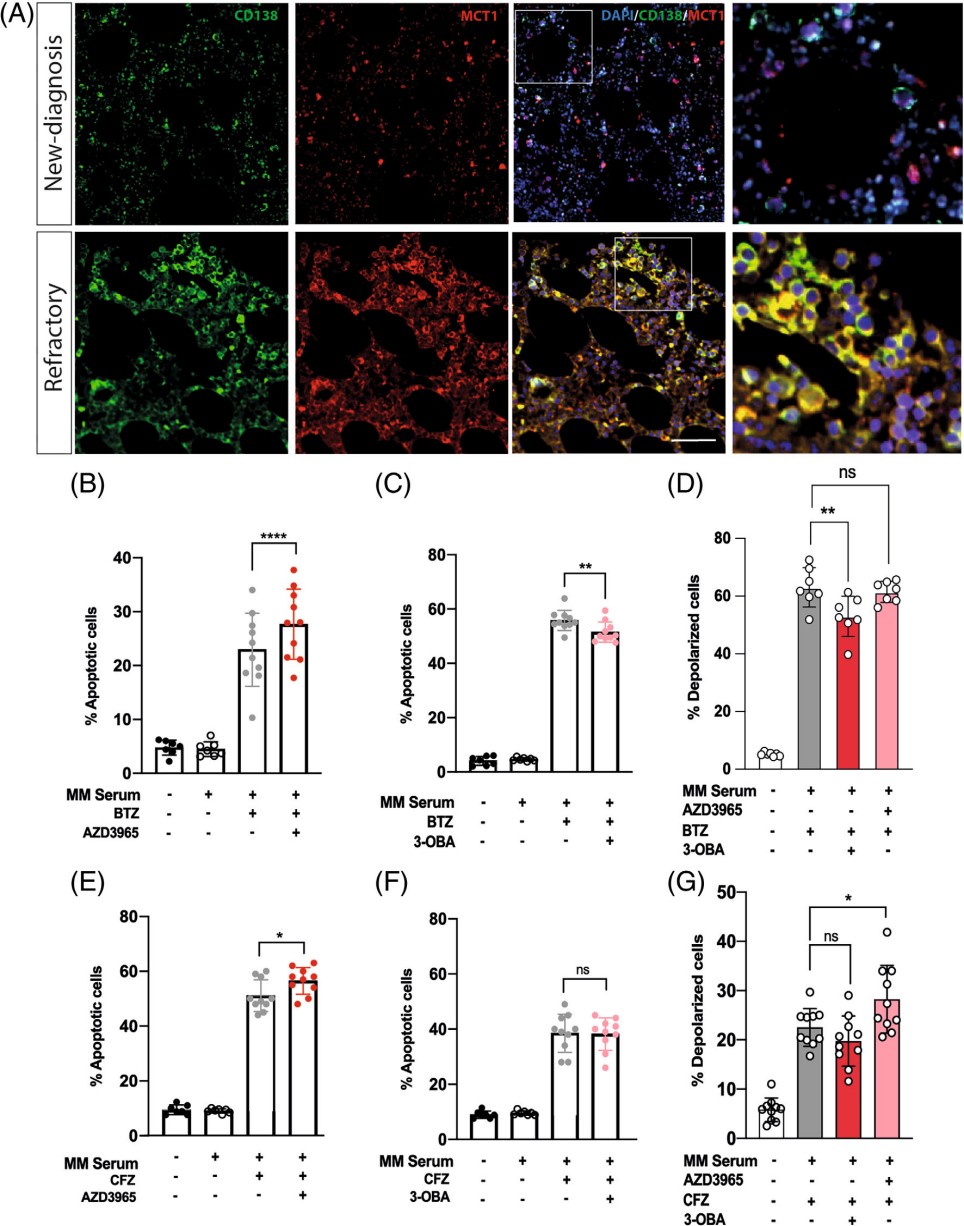
Combination of proteasome inhibitors (PIs) with AZD3965 increases PI‐induced cytotoxicity in vitro. (A) Immunofluorescence images showing co‐localization of MCT1 with CD138^+^ cells in bone marrow biopsy from a multiple myeloma (MM) patient at diagnosis and a refractory one. Scale bar: 50 μm. (B, C) The graphs show the mean values of the percentage of apoptotic cells (annexin‐V and propidium iodide positive) in NCI‐H929 cells cultured in medium supplemented with 20% MM serum after treatment with BTZ or (E, F) CFZ in combination with AZD3965 or 3‐OBA. (D, G) Mitochondrial membrane potential was assessed following DiOC2(3) staining. All the data are presented as means ± *SD* of three independent experiments. **p < 0.01; ****p < 0.0001

Given that the uptake of lactate by myeloma PCs decreased anti‐myeloma efficacy of PIs, we next investigated whether MCT1 is differentially expressed in primary CD138^+^ cells from MM at diagnosis and resistant/refractory patients. Analysing BM biopsy specimens by using immunofluorescence, we found a higher co‐localization of MCT1 within CD138^+^ PCs in resistant/refractory MM compared to patients at diagnosis (Figure 2A).

To elucidate whether the protective effect of lactate was mediated by metabolic activity of lactate or through the activation of GPR81 signalling, NCI‐H929 cells were cultured in medium supplemented with 20% sera from different MM patients (*n* = 10) and treated with BTZ or CFZ alone or in combination with AZD3965, a selective inhibitor of MCT1, or 3‐OBA, an antagonist of GPR81. The optimal dose of AZD3965 was evaluated analysing MM cell viability after exposure to 1, 5, 10, 50 and 100 μM of MCT1 inhibitor. As showed in Figure S2A, 50 and 100 μM AZD3965 significantly decrease cell viability of NCI‐H929. Since we also observed that 10 μM AZD3965 was able to efficiently inhibit the uptake of lactate in myeloma PCs (Figure S2B), this dose was selected for the following experiments. Combination of BTZ or CFZ with AZD3965 caused higher apoptosis rate compared to PI alone (respectively of about 6.3 ± 1.4% for BTZ/AZD3965, *p* < 0.0001 and 5.4 ± 0.9% for CFZ/AZD3965, *p* < 0.05; Figure 2B,E). In contrast to inhibition of MCT1, 3‐OBA decreased the anti‐myeloma effect of BTZ (of about 5.4 ± 2.7% compared to BTZ‐treated cells; *p* < 0.01; Figure 2C) but not of CFZ (Figure 2F). Since it is known that PIs exert their apoptotic effect by disrupting mitochondrial integrity and activity,^19^, ^22^ we also measured mitochondrial polarization status. Compared to BTZ alone, its combination with 3‐OBA significantly reduced the percentage of PI‐induced mitochondrial depolarization (10.1 ± 3.6%; *p* < 0.01; Figure 2D). No difference was observed comparing BTZ/3‐OBA treatment with BTZ alone in MM cells (Figure 2G). However, in these cells mitochondrial depolarization increased significantly of about 4.4 ± 1.2% in PCs treated with CFZ/AZD3965 combination compared to CFZ‐treated cells (*p* < 0.05; Figure 2G). These data suggest that circulating lactate in MM sera protects mitochondria against PI‐induced cytotoxicity and this effect can be counteracted only blocking lactate import using AZD3965. Indeed, circulating lactate reduced PI‐induced mitochondrial depolarization also after blocking lactate/GPR81‐mediated signalling by 3‐OBA.

### 3.3 Acute lactate exposure enhances mitochondrial metabolism in myeloma PCs

**FIGURE 3:**
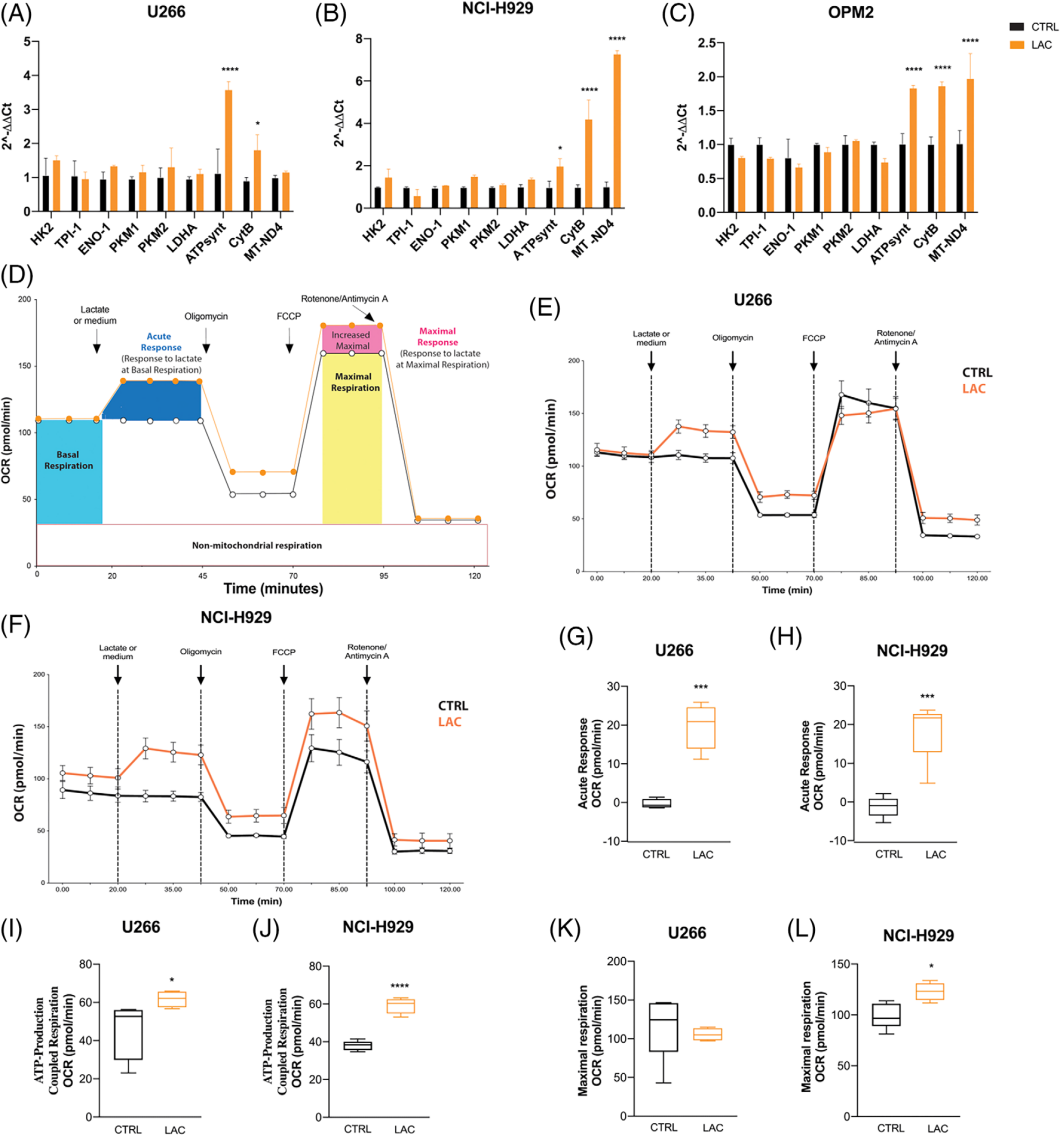
Multiple myeloma cells enhances mitochondrial metabolism after exposure to lactate. (A–C) Gene expression analysis of glycolytic‐ and OXPHOS‐related genes after 3 h lactate exposure. B2M gene was used as housekeeping gene. (D) Schematic principle of the MitoStress test performed. Individual parameters for acute response, proton leak, maximal respiration, non‐mitochondrial respiration and ATP production in U266 (E) and NCI‐H929 (F) cells after injection of lactate. (G–L) Statistical analysis in U266 and NCI‐H929 cell lines of the individual MitoStress test parameters. Measurement was done in three separate experiments with *n* = 5 replicates per condition. All the data are presented as means ± *SD* of three independent experiments. *p < 0.05; **p < 0.01; ***p < 0.001; ****p < 0.0001

The finding that lactate protect against PIs led us to examine its metabolic effects in MM cells. We first evaluated the expression of glycolytic‐ and oxidative phosphorylation (OXPHOS)‐related genes after 3 h from exposure to high lactate concentration. A significant up‐regulation of ATPsynt and CytB mRNA was observed in HMCLs (Figure 3A–C): moreover, OPM2 and NCI‐H929 also increased the expression of MT‐ND4 (*p* < 0.0001 compared to control). As metabolic processes in the mitochondria produce ROS, we subsequently analysed the amount of mROS by flow cytometry using a probe that selectively detects mitochondrial superoxide. Lactate treatment significantly increased the percentage of mROS both in U266 and NCI‐H929 cell lines already after 3 h (of about 69.8 ± 14.4% and 43.7 ± 9%; *p* < 0.0001 and *p* < 0.001, respectively; Figure S3A,B). Interestingly, the same trend was confirmed also after 24 h (41.9 ± 20.8% and 24.4 ± 9%; *p* < 0.01 and *p* < 0.05 compared to respective control). To confirm that lactate can be uptaken and used as a mitochondrial respiratory substrate by myeloma PCs, we next evaluated the acute effect of lactate by metabolic profiling using Seahorse platform. OCR was quantified for 25 min immediately after lactate injection to analyse the effect of the metabolite on basal respiration (acute response). After 25 min, oligomycin was injected to estimate mitochondrial ATP production and subsequently FCCP (carbonyl cyanide‐ptrifluoromethoxyphenylhydrazone) was used to provide maximal mitochondrial respiration (Figure 3D). Compared to cells injected with medium only, MM cells showed an increase of the basal respiration after lactate injection (Figure 3E,F) with a significant acute response of about 19.9 ± 4.6 and 19.8 ± 5 OCR pmol/min respectively for U266 and NCI‐H929 (*p* < 0.001; Figure 3G,H). After injection of oligomycin, both MM cell lines exposed to lactate produce higher mitochondrial ATP than control cells (*p* < 0.05 and *p* < 0.0001 for U266 and NCI‐H929 cells; Figure 3I,J). NCI‐H929 cells also exhibited a significant increase of the maximal respiration (*p* < 0.01 compared to control; Figure 3L). No difference was observed in U266 cells (Figure 3K).

### 3.4 Chronic exposure to high concentration of lactate rewires metabolic processes affecting myeloma cell growth

**FIGURE 4:**
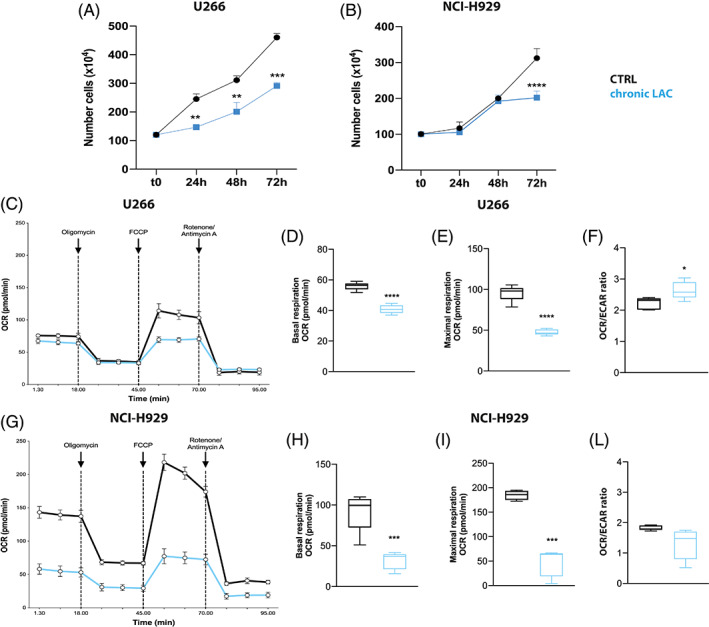
Metabolic adaptation of myeloma plasma cells to high concentration of lactate. (A, B) Cell proliferation assessed in multiple myeloma cell lines grown in medium control or supplemented with lactate for 72 h. Graphs show the mean of cell number ± *SD* evaluated using EVE™ automated cell counter. (C) Evaluation of OCR in U266 and (G) NCI‐H929 after 72 h culture with medium control or supplemented with lactate. (D–F) Quantification of U266 and (H–L) NCI‐H929 cells MitoStress test parameters. Measurement was done in three separate experiments with *n* = 5 replicates per condition. All the data are presented as means ± *SD* of three independent experiments. *p < 0.05; **p < 0.01; ***p < 0.001; ****p < 0.0001

To better dissect the effects of high concentrations of lactate in tumour microenvironment, we cultured myeloma PCs in medium supplemented with lactate for 3 days. U266 cells growth in presence of lactate showed a marked reduction in proliferation compared to control (*p* < 0.001, *p* < 0.001 and *p* < 0.001 after 24, 48 and 72 h; Figure 4A). The growth of NCI‐H929 cell line was slower only after 72 h (*p* < 0.0001 vs. control; Figure 4B).

Next, we examined whether the reduced proliferative capacity of PCs after chronic exposure to lactate might be associated to changes in cellular metabolism. After 72 h, we found that myeloma cells chronically growth in presence of high lactate concentration were unable to increase oxygen consumption (Figure 4C,G). Compared to control cells, both U266 and NCI‐H929 exhibited lower basal (*p* < 0.0001 and *p* < 0.001; Figure 4D,H) and maximal respiration (*p* < 0.0001 and *p* < 0.001; Figure 3E,I). Also extracellular acidification rate (ECAR) was significantly decreased in PCs growth in medium supplemented with lactate (*p* < 0.0001; Figure S4A,B). However, showing an opposite trend compared to NCI‐H929 (Figure 4L), U266 cells maintained increased OCR/ECAR ratio (*p* < 0.05; Figure 4F) indicating that these cells have higher OCR and lower ECAR activities because they rely on OXPHOS for energy production.

### 3.5 Lactate regulates tumour immunosuppression in MM microenvironment as metabolic mediator

**FIGURE 5:**
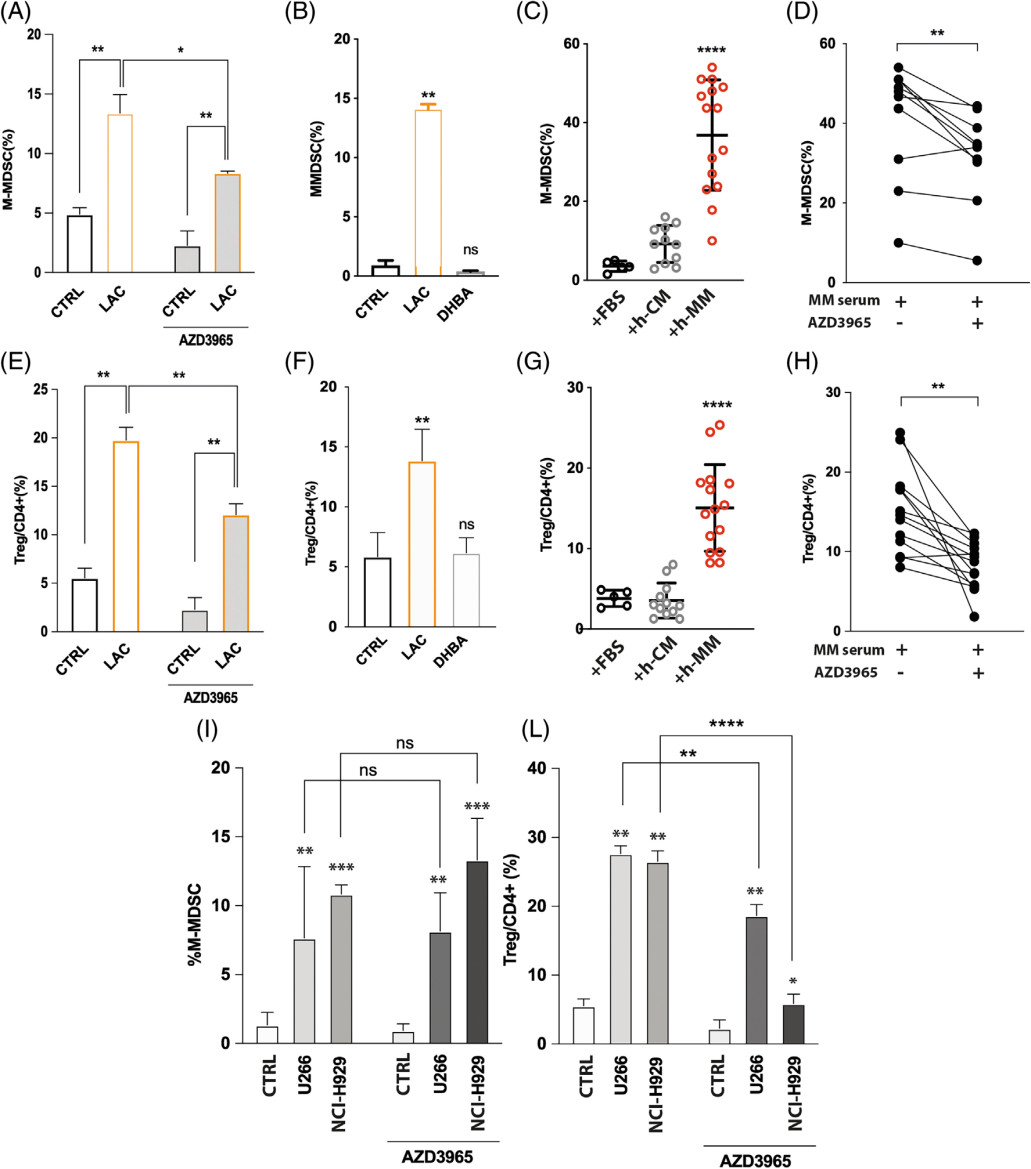
High lactate concentrations promote M‐MDSCs and T‐reg expansion in multiple myeloma (MM) microenvironment. (A) Evaluation of the percentage of M‐MDSC and (E) Treg in peripheral blood mononucleated cells (PBMCs) after exposure to lactate alone or in combination with AZD3965. (B) 3,5 DHBA does not induce expansion of M‐MDSCs and (F) Treg. (C) Analysis of M‐MDSCs and (G) Treg percentages after culturing PBMCs in h‐CM or h‐MM. (D) Evaluation of AZD3965 effects on circulating lactate‐induced M‐MDSCs and (H) Treg expansion. (I–L) M‐MDSCs and Treg analysis in PBMCs after co‐culture with HMCLs in presence or not of AZD3965. Data are presented as means ± *SD* of three independent experiments. *p < 0.05; **p < 0.01; ***p < 0.001; ****p < 0.0001

Finally, we explored the effects of high concentration of lactate in promoting immune escape mechanisms in myeloma microenvironment. First, we exposed healthy PBMCs to lactate w/o AZD3965. After 48 h we observed a significant increase of the amount of M‐MDSCs (CD14+/HLA‐DR^−^) and Treg (CD4^+^/CD25^+^/FOXP3^+^) percentages (respectively of about 8.5 ± 2.1% and 14.2 ± 0.3% compared to control; *p* < 0.01; Figure 5A,E). No changes were observed evaluating the percentage of granulocytic like MDSCs (data not showed). AZD3965 exposure significantly decreased their amount respectively of 5 ± 1.7% and 7.7 ± 2.4% in respect of lactate treatment alone (*p* < 0.05 and *p* < 0.01; Figure 5A,E). To assay the involvement of lactate/GPR81 pathway in inducing the expansion of immune‐suppressive populations, we exposed PBMCs to 3,5‐DHBA, a selective agonist of GPR81.^35^ Data showed that 3,5 DHBA treatment did not induce any significant increase neither of M‐MDSCs nor Treg (Figure 5B,F), demonstrating that lactate did not promote immune‐suppressive cell expansion in a hormone‐like manner. To better define the immunosuppressive role of lactate in myeloma microenvironment, PBMCs were cultured in medium conditioned with 20% sera from healthy controls (healthy conditioned medium, h‐CM; *n* = 11) or MM patients at diagnosis (MM conditioned medium, MM‐CM; *n* = 15) for 48 h. A significant increase of about 27.9 ± 11.9% for M‐MDSCs and 13.1 ± 4.3% for Treg was found in PBMCs cultured with MM‐CM compared to the same cells growth in h‐CM (*p* < 0.0001; Figure 5C,G). Adding AZD3965 to cells cultured with MM‐CM, we found a significant decrease of both M‐MDSCs and Treg of about 10.5 ± 6% and 7.5 ± 6.2% compared to PBMCs cultured in presence of MM sera only (*p* < 0.01; Figure 5D,H).

As a last step, we investigated whether myeloma PCs could be directly involved in the expansion of immunosuppressive subsets through the release of lactate. As expected, co‐culturing PBMCs with MM cell lines both M‐MDSCs and Treg subpopulations resulted significantly increased after 48 h (Figure 5I,L), but only Treg expansion was reduced by AZD3965 (of about 9 ± 2.7% and 20.6 ± 2.9% for PBMCs co‐cultured respectively with U266 and NCI‐H929 compared to co‐culture without MCT1 inhibitor; *p* < 0.01 and *p* < 0.0001; Figure 5L). Collectively, these results suggest that the highest levels of circulating lactate in MM patients favour expansion of Treg and MDSCs and this metabolite is a mediator of Treg activation induced by tumour PCs.

## 4 DISCUSSION

Metabolic reprogramming, a hallmark of human tumours, represent an important pathophysiological mechanism in MM progression. In this context, metabolic features of tumour PCs are typical of the Warburg effect with increased lactate production,^36^ but at the same time, MM cells can use the reverse Warburg effect taking up lactate from tumour microenvironment by using MCT1.^24^, ^37^ To this regard, both MCT1 and MCT4 show highest substrate specificity for lactate; however, when MCT1 is expressed, it represents the first regulator of cellular lactate flux.^38^ Consistently, Walters and colleagues analysed expression of lactate transporters on MM cells and found an overexpression of MCT1 in MM PCs compared to MGUS.^39^ Moreover, the authors have also demonstrated that MCT1 has a predominant role in lactate flux compared to MCT4 and plays an important role in regulating tumour growth.^39^ Here, we report that, when compared to healthy subjects, MM patients have higher levels of circulating lactate which are, however, increased also in MGUS and SMM patients. It is therefore conceivable that myeloma PCs become more responsive to lactate and its flux, in accordance with the previously observation that MCT1 expression increases steadily with the progression state of PC disorders.^39^, ^40^ Our data confirmed that MCT1 expression is higher than MCT4 in HMCLs. Under physiological conditions, MCT1 is preferentially expressed in normoxic cells, whereas hypoxia‐inducible MCT4 has been primarily reported to be expressed in glycolytic/hypoxic cells.^41^ Exposing MM cells to high levels of lactate resulted in a significant increase of both MCTs and the lactate receptor GPR81 in all tested HMCLs. Only NCI‐H929 showed a decrease of MCT4 expression after 24 h. As recently it has been reported that lactate can confer chemo‐resistance in cancer,^42^ we found a significant reduction of the apoptotic effect of BTZ and CFZ in MM cells grown in medium with high concentration of lactate, especially in U266 cells.

Consistently with previously reports,^40^ our results showed higher levels of MCT1 in CD138^+^ PCs from resistant/refractory patients in respect to patients at diagnosis. Recently, MCT1 has been identified as a predictive marker for the efficacy of lenalidomide maintenance therapy in MM patients with a reduced PFS and OS in patients with higher expression of the lactate transporter.^40^ Furthermore, the overexpression of MCT1 protects myeloma PCs from the antimyeloma activity of lenalidomide. One approach to take advantage of the metabolic vulnerability of cancer cells has been through the development of AZD3965, an orally bioavailable inhibitor of MCT1, currently under phase I clinical trial (NCT01791595). Our data indicate that combination of PI with AZD3965 can be synergistic, increasing tumour PC death. Indeed, pharmacological inhibition of MCT1 prevented the import of lactate circulating in MM sera overcoming its metabolic protective effects. Inhibition of lactate/GPR81‐mediated signalling by 3‐OBA reduced the efficacy of BTZ, but not of CFZ, in presence of high levels of circulating lactate, probably augmenting lactate metabolic activity. Corresponding to this hypothesis, combination of 3‐OBA with PI decreased the drug‐induced mitochondrial depolarization; on the contrary, AZD3965/CFZ treatment increased this effect.

Lactate is taken up and used as a mitochondrial respiratory substrate due to mitochondrial lactate carriers and a mitochondrial LDH localized in the matrix/inner membrane component.^43^, ^44^ Lactate is then oxidized to pyruvate and then converted to acetyl‐CoA through mitochondrial lactate oxidation complex.^45^ Our data demonstrated that myeloma PCs exposed to high lactate concentration upregulated OXPHOS‐related genes and increased the amount of mROS. The lactate‐induced oxidative metabolism was then confirmed by the higher basal respiration, mitochondrial ATP production and maximal respiratory capacity found in PCs after exposure to lactate. However, growing MM cells in medium supplemented with lactate resulted in a gradual decrease in cell growth associated to the inability to increase oxygen consumption. Both basal and maximal respiration were significantly decreased, but the OCR/ECAR ratio remained higher in U266 cells growth in medium with high lactate concentration in respect of control, suggesting a higher contribution of mitochondrial respiration versus glycolysis to energy generation. This metabolic switch, resulting from chronic exposure to lactate, is essential to limiting the anti‐myeloma effect of PIs. The proteasome is involved in the regulation of cellular metabolism; indeed, inhibition of proteasome activity leads to a global metabolic response.^19^, ^22^ To survive proteasome inhibition, myeloma PCs change their metabolism, mitochondria and endoplasmic reticulum to redistribute cellular resource and reduce their metabolic fitness.^7^ OXPHOS stimulation takes part to the mechanisms with which PCs adapt metabolically and maintain bio‐energetic plasticity.^19^, ^46^, ^47^, ^48^ Notably, this OXPHOS‐protective anti myeloma effect is in agreement with the significant decrease of PI‐induced apoptosis observed in myeloma PCs grown in medium with high concentration of lactate.

Interestingly, in addition to its uptake by tumour cells, lactate also induces immunosuppression in the TME.^49^, ^50^ As a consequence of the high extracellular lactate levels, the expression of Foxp3, the key transcription factor for Treg function, increases. Foxp3 stimulates oxidation of lactate to fuel mitochondrial activity, providing Treg with a metabolic advantage in high‐lactate conditions.^23^ Moreover, lactate downregulates FIP200 expression leading to naïve T cell apoptosis and autophagy impairment,^51^ decreases the cytolytic functions of CD8+ T cells^52^ and cytotoxic activity of NK.^53^ Based on this, we evaluated the immunosuppressive effects of lactate in MM microenvironment, in particular on expansion of Treg and MDSCs which are significantly increased in MM microenvironment.^54^, ^55^, ^56^, ^57^ Furthermore, MDSCs contribute to Treg differentiation, suppress T cell proliferation, promote angiogenesis and proliferation of myeloma PCs and even differentiate themselves into functional osteoclasts.^54^, ^58^, ^59^ Exposing healthy PBMCs to high lactate concentration, we found not only an increase of the percentage of Treg but also of M‐MDSCs. This lactate‐mediated expansion was not dependent by lactate/GPR81 pathway as demonstrated by treatment of PBMCs with the selective agonist of GPR81, DHBA. This data was consistent with the observation that a significant reduction of both Treg and M‐MDSC subtypes was found after blocking MCT1. To demonstrate the involvement of lactate in Treg and M‐MDSCs expansion in MM, we then cultured PBMCs in presence of serum from HC or MM patients. In our experimental conditions, both immunosuppressive subsets increased only in presence of MM sera and the high levels of circulating lactate are in part responsible of these subset expansions as demonstrated by the significant reduction of the percentage of Treg or M‐MDSCs after adding AZD3965. The expansion of Treg and M‐MDSCs can be directly regulated by tumour PCs.^60^, ^61^, ^62^ Therefore, we sought to examine whether tumour release of lactate may be involved. We found a significant decrease of the percentage of tumour‐induced Treg, but not of M‐MDSCs, after blocking MCT1. Taken all together, these results suggest that the highest levels of circulating lactate in MM patients favour expansion of Treg and MDSCs and this metabolite is a direct mediator of tumour PC‐induced Treg activation.

**FIGURE 6:**
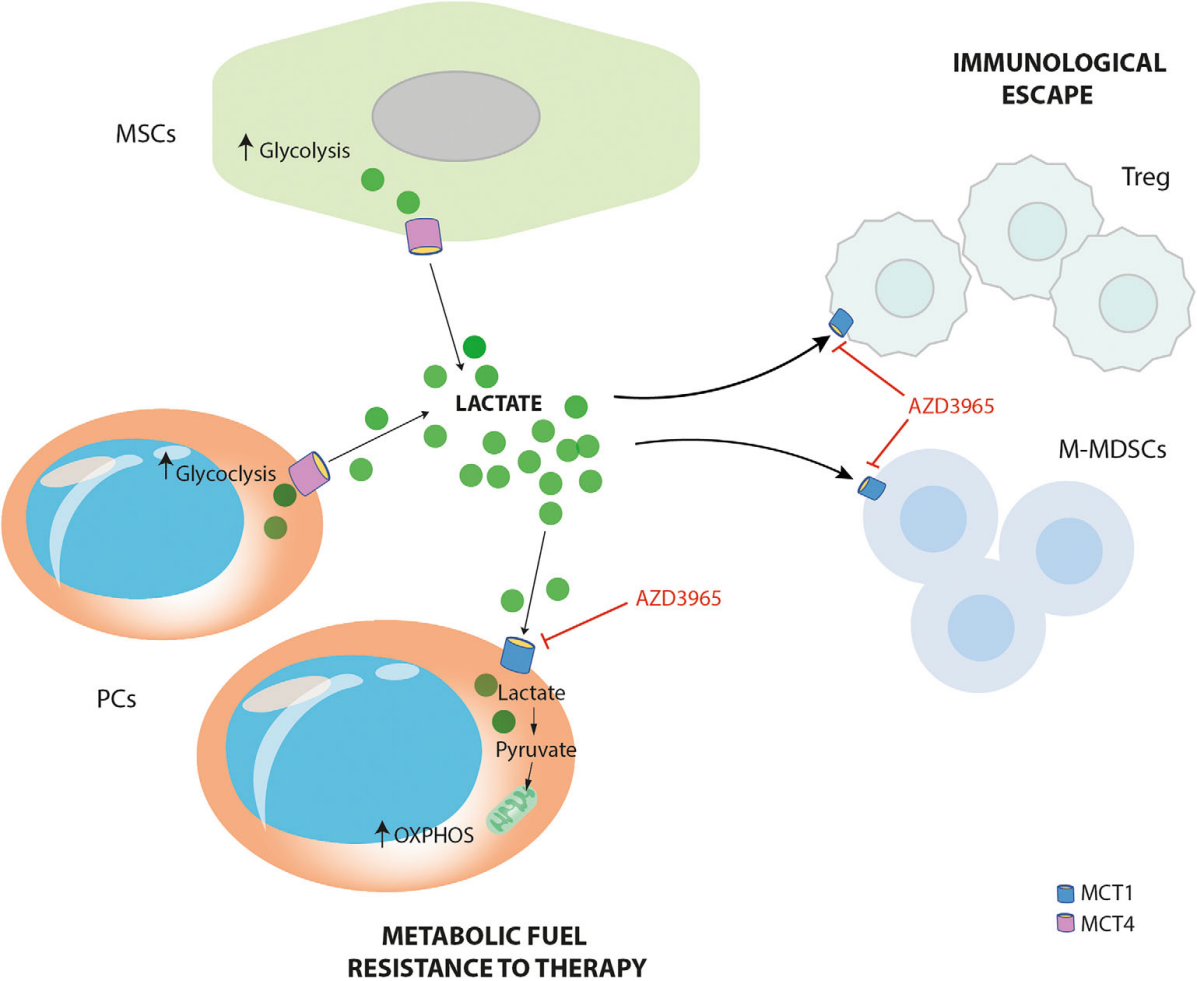
Lactate trafficking modulates tumour microenvironment in multiple myeloma

In conclusion, in the current study we documented that high concentration of lactate in MM patients change PC bioenergetics favouring mitochondrial metabolism and resistance to PIs. Accordingly, blocking MCT1, but not the hormone‐like activity of lactate, increased the anti‐myeloma effects of PIs. Moreover, our data revealed that lactate is not only a metabolite, but also an important regulator of immune response establishing an immunosuppressive pro‐tumoral microenvironment in MM patients which can weaken the therapeutic effect of immunotherapy (Figure 6). Together, these results established that metabolic targeting approaches inhibiting lactate trafficking in TME could represent a potential key strategy for anti‐cancer therapy in MM.

## AUTHOR CONTRIBUTIONS

Alessandro Barbato, Cesarina Giallongo and Daniele Tibullo contributed to conceptualization; contributed to project administration; Marco Lolicato, Giacomo Lazzarino, Nunziatina Parrinello, Vittorio Del Fabro, Paolo Fontana, M'hammed Aguennoz, Giovanni Li Volti, Giuseppe A. Palumbo, Alessandra Romano, Grazia Scandura, Saoca Concetta, Tatiana Zuppelli, Sebastiano Giallongo contributed to methodology; Alessandro Barbato, Cesarina Giallongo, Alessandra Romano, Grazia Scandura, Saoca Concetta, Tatiana Zuppelli, Sebastiano Giallongo contributed to investigation; Alessandro Barbato, Cesarina Giallongo, Paolo Fontana, Giuseppe A. Palumbo, Alessandra Romano, Daniele Tibullo contributed to formal analysis; Francesco Di Raimondo, Paolo Fontana, Daniele Tibullo, Cesarina Giallongo, Giovanni Li Volti contributed to supervision; Cesarina Giallongo, Daniele Tibullo, Sebastiano Giallongo, Francesco Di Raimondo, contributed to writing—original draft.

## FUNDING INFORMATION

The research leading to these results has received funding from the AIRC under IG 2018 ‐ ID. 22131 P.I. Di Raimondo Francesco. This study was supported by the Piano di Incentivi per la ricerca di Ateneo 2020/2022 Linea di intervento 2 (Prof. Francesco Di Raimondo). Cesarina Giallongo was supported by the PON AIM R&I 2014–2020‐E68D19001340001. This study was supported in part by Associazione Italiana contro le Leucemie‐Linfomi e mieloma (A.I.L.) section of Catania, and Fondazione Catanese per lo Studio delle Malattie Neoplastiche del Sangue (FON.CA.NE.SA.).

## CONFLICT OF INTEREST

The authors declare no conflicts of interest.

## CONSENT FOR PUBLICATION

All authors read and approved the final manuscript.

## Supporting information


**Figure S1.** (A) Correlation between lactate levels and the amount of LDH‐A in PB of patients with plasma cell disorders. (B) Gene expression analysis of GPR81 after 3 h lactate exposure. B2M gene was used as housekeeping gene. Data are presented as means ± *SD* of three independent experiments. ***p < 0.001.
**Figure S2.** (A) NCI‐H929 cells (1 × 10^5^) were seeded in a 96‐well plate and incubated with different concentration of AZD3965. After 24 h the XTT cell viability kit (Cell signalling tech. 9095) was added to the plate and cells were incubated for 4 h. The absorbance at 450 nm was measured. (B) Evaluation of intracellular lactate in NCI‐H929 after 30 min from exposure to 20 mM lactate in presence or not of 1 or 10 μM AZD3965. Data are presented as means ± *SD* of three independent experiments. **p < 0.01; ***p < 0.001; ****p < 0.0001.
**Figure S3.** (A,C) Flow cytometric analysis of mROS. Data are expressed as % of MitoSOX™ Red positive cells presented as means ± *SD* of three independent experiments. (B, D) Representative dot plots illustrating mROS in U266 and NCI‐H929 cells after exposure to lactate for 30 min, 1 h, 3 h and 24 h. *p < 0.05; **p < 0.01; ***p < 0.001, ****p < 0.0001.
**Figure S4.** (A‐B) Statistical analysis in U266 and NCI‐H929 cell lines of ECAR. Measurement was done in three separate experiments with *n* = 5 replicates per condition. ****p < 0.0001.

## Data Availability

The data that support the findings of this study are available from the corresponding authors upon reasonable request.
