# Supplementary material for: Lactate trafficking inhibition restores sensitivity to proteasome inhibitors and orchestrates immuno‐microenvironment in multiple myeloma
Source: Cell Prolif. 2023 Feb 15;56(4):e13388. doi: 10.1111/cpr.13388 (PMC10068934; doi:10.1111/cpr.13388)
Supplement: Supplementary file 1 — Figure S1. (A) Correlation between lactate levels and the amount of LDH‐A in PB of patients with plasma cell disorders. (B) Gene expression analysis of GPR81 after 3 h lactate exposure. B2M gene was used as housekeeping gene. Data are presented as means ± SD of three independent experiments. ***p < 0.001. Figure S2. (A) NCI‐H929 cells (1 × 10^5^) were seeded in a 96‐well plate and incubated with different concentration of AZD3965. After 24 h the XTT cell viability kit (Cell signalling tech. 9095) was added to the plate and cells were incubated for 4 h. The absorbance at 450 nm was measured. (B) Evaluation of intracellular lactate in NCI‐H929 after 30 min from exposure to 20 mM lactate in presence or not of 1 or 10 μM AZD3965. Data are presented as means ± SD of three independent experiments. **p < 0.01; ***p < 0.001; ****p < 0.0001. Figure S3. (A,C) Flow cytometric analysis of mROS. Data are expressed as % of MitoSOX™ Red positive cells presented as means ± SD of three independent experiments. (B, D) Representative dot plots illustrating mROS in U266 and NCI‐H929 cells after exposure to lactate for 30 min, 1 h, 3 h and 24 h. *p < 0.05; **p < 0.01; ***p < 0.001, ****p < 0.0001. Figure S4. (A‐B) Statistical analysis in U266 and NCI‐H929 cell lines of ECAR. Measurement was done in three separate experiments with n = 5 replicates per condition. ****p < 0.0001. [file CPR-56-e13388-s001.docx]

**Supplementary Figures**


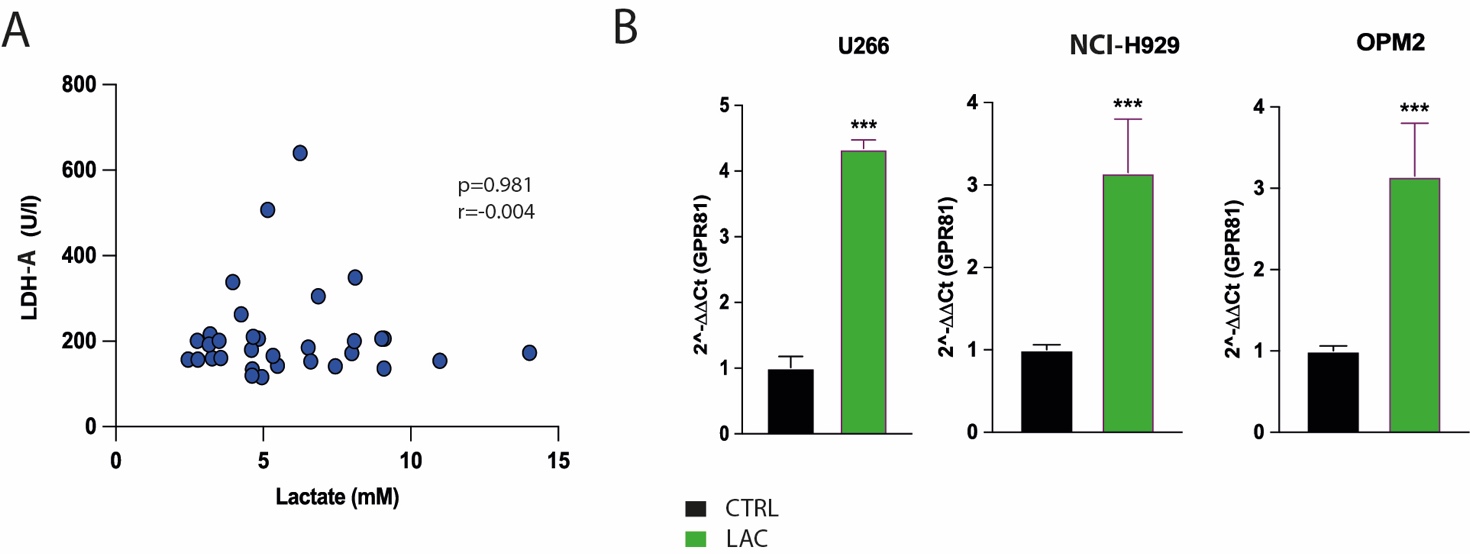


**Supplementary Figure 1. (A)** Correlation between lactate levels and the amount of LDH-A in PB of patients with plasma cell disorders. **(B)** Gene expression analysis of GPR81 after 3h lactate exposure. B2M gene was used as housekeeping gene. Data are presented as means ± SD of three independent experiments. ***p<0.001


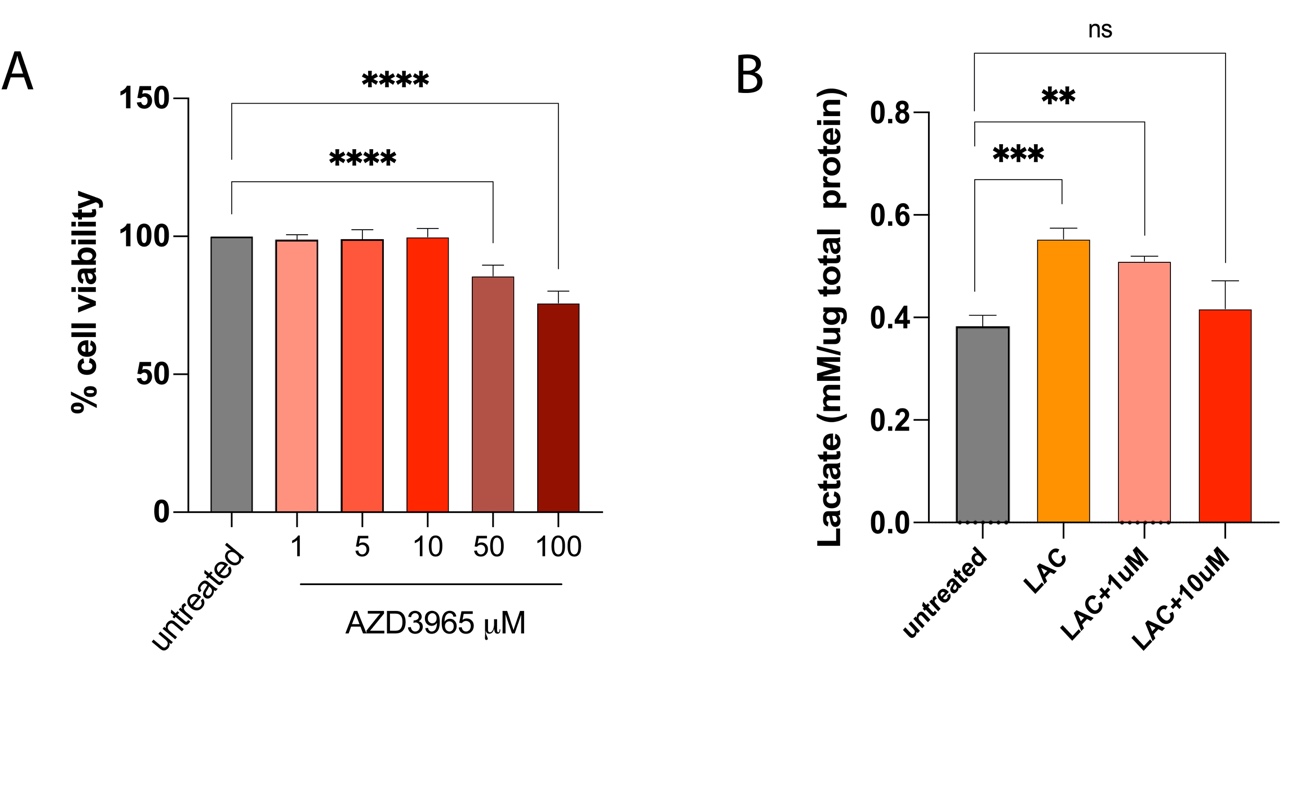


**Supplementary Figure 2. (A)** NCI-H929 cells (1x10^5^) were seeded in a 96-well plate and incubated with different concentration of AZD3965. After 24h the XTT cell viability kit (Cell signaling tech. 9095) was added to the plate and cells were incubated for 4h. The absorbance at 450 nm was measured. **(B)** Evaluation of intracellular lactate in NCI-H929 after 30 min from exposure to 20 mM lactate in presence or not of 1 or 10 µM AZD3965. Data are presented as means ± SD of three independent experiments. **p<0.01; ***p<0.001; ****p<0.0001


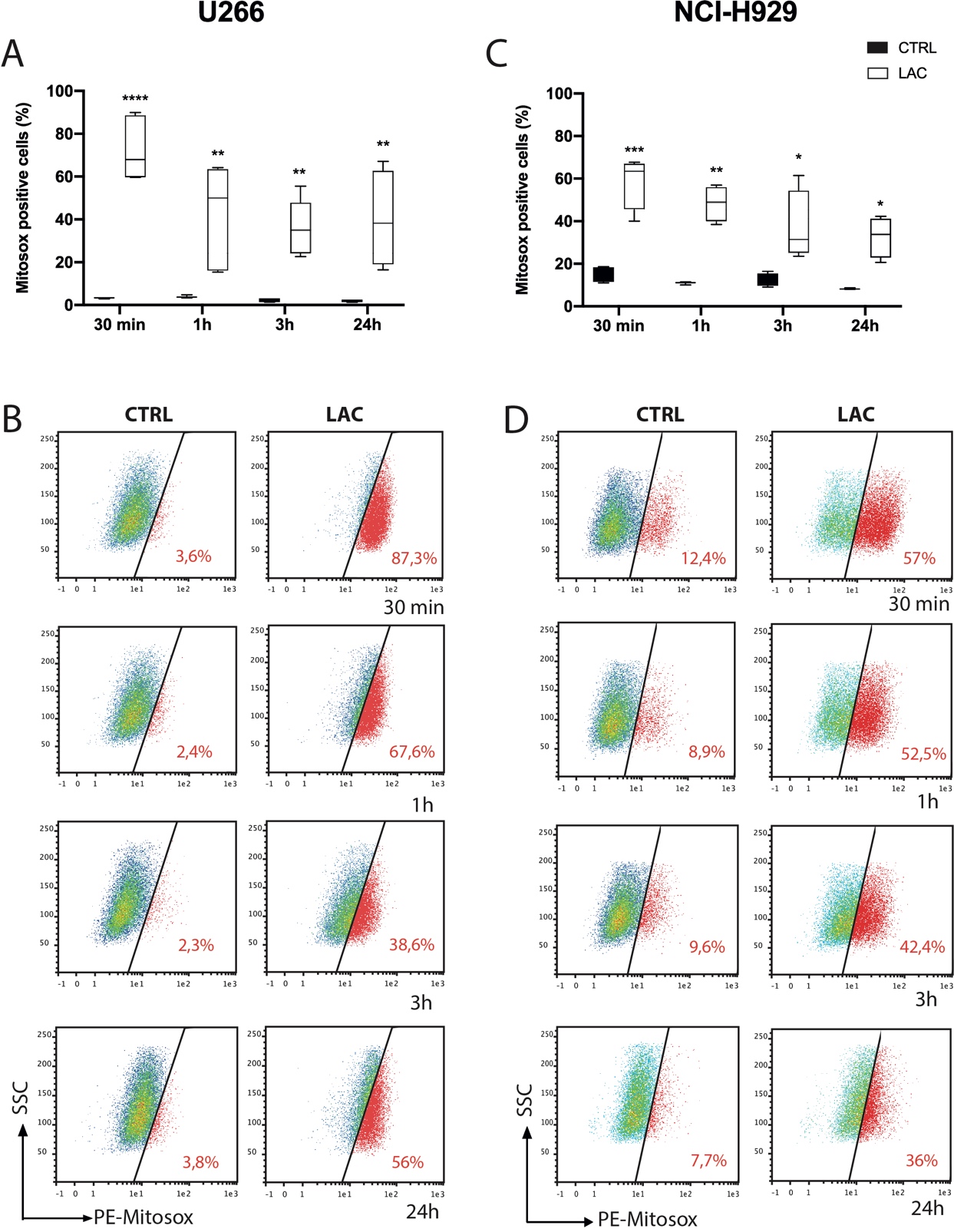


**Supplementary Figure 3. (A,C)** Flow cytometric analysis of mROS. Data are expressed as % of MitoSOX™ Red positive cells presented as means ± SD of three independent experiments. **(B,D)** Representative dot plots illustrating mROS in U266 and NCI-H929 cells after exposure to lactate for 30 min, 1h, 3h and 24h. *p<0.05; **p<0.01; ***p<0.001, ****p<0.0001.


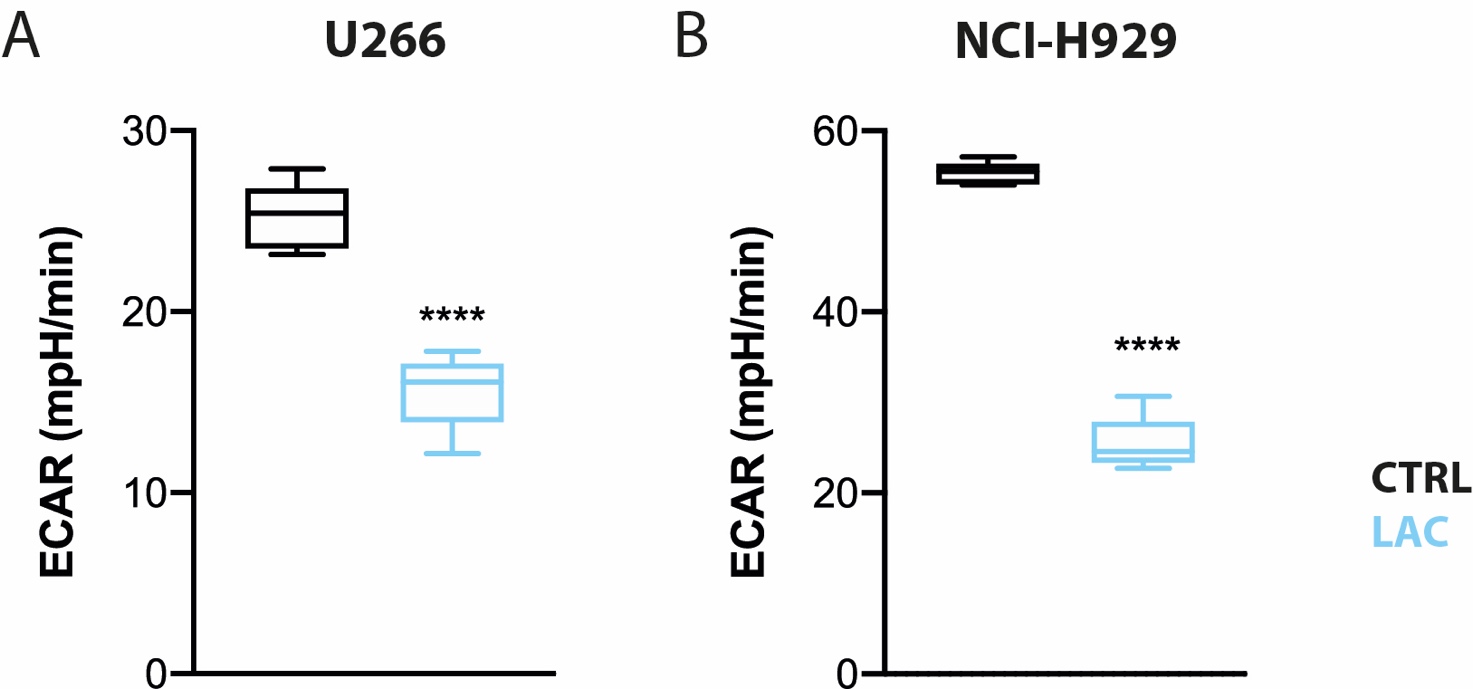


**Supplementary Figure 4. (A-B)** Statistical analysis in U266 and NCI-H929 cell lines of ECAR. Measurement was done in three separate experiments with n=5 replicates per condition. ****p<0.0001
